# Tracking down the path of memory: eye scanpaths facilitate retrieval of visuospatial information

**DOI:** 10.1007/s10339-015-0690-0

**Published:** 2015-08-11

**Authors:** Agata Bochynska, Bruno Laeng

**Affiliations:** Department of Language and Literature, Norwegian University of Science and Technology, Trondheim, Norway; Department of Psychology, University of Oslo, Oslo, Norway

**Keywords:** Visuospatial memory, Eye movements, Scanpath, Eye fixations, Eye-tracking

## Abstract

Recent research points to a crucial role of eye fixations on the same spatial locations where an item appeared when learned, for the successful retrieval of stored information (e.g., Laeng et al. in Cognition 131:263–283, 2014. doi:10.1016/j.cognition.2014.01.003). However, evidence about whether the specific temporal sequence (i.e., scanpath) of these eye fixations is also relevant for the accuracy of memory remains unclear. In the current study, eye fixations were recorded while looking at a checkerboard-like pattern. In a recognition session (48 h later), animations were shown where each square that formed the pattern was presented one by one, either according to the same, idiosyncratic, temporal sequence in which they were originally viewed by each participant or in a shuffled sequence although the squares were, in both conditions, always in their correct positions. Afterward, participants judged whether they had seen the same pattern before or not. Showing the elements serially according to the original scanpath’s sequence yielded a significantly better recognition performance than the shuffled condition. In a forced fixation condition, where the gaze was maintained on the center of the screen, the advantage of memory accuracy for same versus shuffled scanpaths disappeared. Concluding, gaze scanpaths (i.e., the order of fixations and not simply their positions) are functional to visual memory and physical reenacting of the original, embodied, perception can facilitate retrieval.

## Introduction

Recently, a growing number of studies have pointed to the possibility of a functional role of eye movements in visuospatial memory. Research shows that eyes tend to re-fixate to the same locations as during encoding (e.g., Valuch et al. 2013; Spivey and Geng 2001; Laeng et al. 2014) and that spatial locations of eye fixations could be used as cues in memory retrieval (e.g., Hebb 1968; Hochberg 1968; Neisser 1967; Winograd and Church 1988). Additionally, in several studies a general advantage for memory performance at recognition was observed when the gaze revisited the same locations as during encoding (e.g., Foulsham and Kingstone 2012; Foulsham and Underwood 2008; Hollingworth and Henderson 2002; Holm and Mäntylä 2007; Johansson and Johansson 2014; Laeng et al. 2014; Mäntylä and Holm 2006; Stark and Ellis 1981; Underwood et al. 2009; Valuch et al. 2013). Moreover, some researchers noticed that perturbating spontaneous eye movements could disrupt memory of details about the object (Johansson and Johansson 2014; Laeng et al. 2014; Mäntylä and Holm 2006).

Importantly, this gaze behavior is not triggered by salient features in the picture in a bottom-up fashion, as shown in the studies that investigated visual imagery (e.g., Johansson et al. 2012; Laeng et al. 2014; Laeng and Teodorescu 2002). Inspired by the Brand and Stark’s (1997) observations and their idea behind the Scanpath Theory, Laeng and Teodorescu (2002) conducted a study where they observed that reenactment of the similar scanpath can provide a cue for memory about a particular object or scene. Thus, they proposed that eye movements are functional, which is consistent with the idea that the perception is active and cognition is embodied. In the current study, we tested directly whether the serial order of eye fixations, recorded first when learning a set of checkerboard-like patterns, played a beneficial role in a recognition session (48 h later), when these were reenacted in the same, idiosyncratic, temporal sequence in which they were originally viewed. We hypothesized that a shuffled sequence of fixations, though still in their correct positions, would lead to a decrease in memory accuracy or in its efficiency (i.e., a lengthening of response times).

## Methods

### Participants

Twenty-eight right-handed participants (17 females) with normal or corrected to normal vision were recruited to participate in a study of visual memory (mean age 26.35 years, SD = 6.45).

### Procedure

**Fig. 1:**
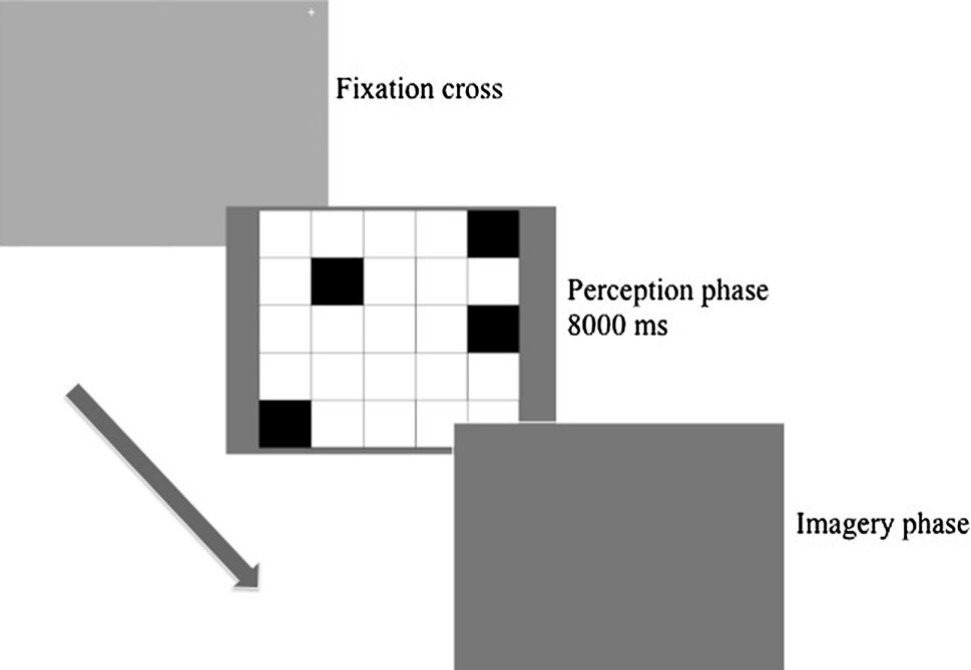
Illustration of the encoding session procedure with perception and imagery phase

Eye monitoring was obtained with an iView Remote Eye-Tracking Device (R.E.D.) from Senso-Motoric Instruments (SMI, Berlin, Germany) and *iView 3.0*^*®*^*Experiment Center* software was used for data collection and stimulus presentation. Every testing session was preceded with a standard calibration procedure. Testing took place in the Cognitive Laboratories at the Institute of Psychology, University of Oslo. Every participant took part in two testing sessions (encoding and recognition) with 48 h delay. In the first session participants were looking at 32 images of 5 × 5 grids resembling checkerboards where 4 black squares formed a random pattern and the rest remained white. Figure 1 represents the details of procedure in the encoding session. Participants were instructed to memorize the patterns as accurately as possible. Every time the picture disappeared from the screen they were asked to imagine the pattern once again and press the space-bar key whenever they were ready to see the next image.

**Fig. 2:**
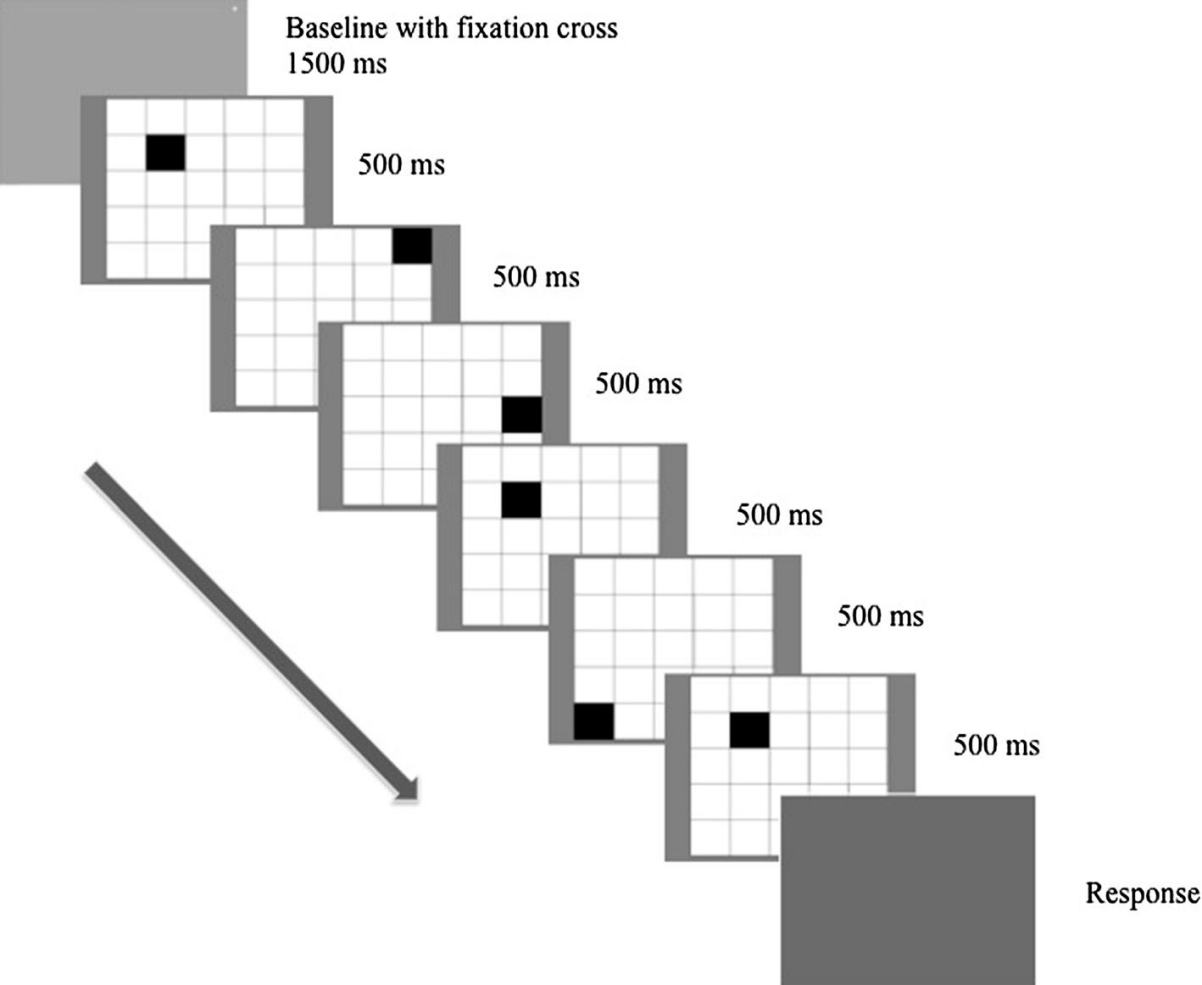
Illustration of the recognition session procedure with baseline, serial presentation of the pattern and response display

Registered eye sequences from the first session determined the stimuli presentation for the second session, which was individually adjusted for every participant. Instead of static images, participants were recognizing the patterns while looking at animations where black squares appeared one after another in their correct positions but in two types of sequences—either according to the original sequence of participant’s eye movements from the first session or in a shuffled sequence (Fig. 2). Additionally, patterns from the encoding session were mixed with 32 additional (novel) patterns presented in random sequences. When the picture disappeared, participants pressed ‘M’ key on the keyboard for ‘old’ patterns or ‘Z’ key for ‘novel’ patterns. To additionally manipulate the involvement of the eye movements, recognition session was divided into two separate blocks—free viewing (participants were allowed to freely follow the appearing squares) and forced fixation (participants were asked to fixate their gaze in the middle of the screen).

## Results

### Behavioral results

**Fig. 3:**
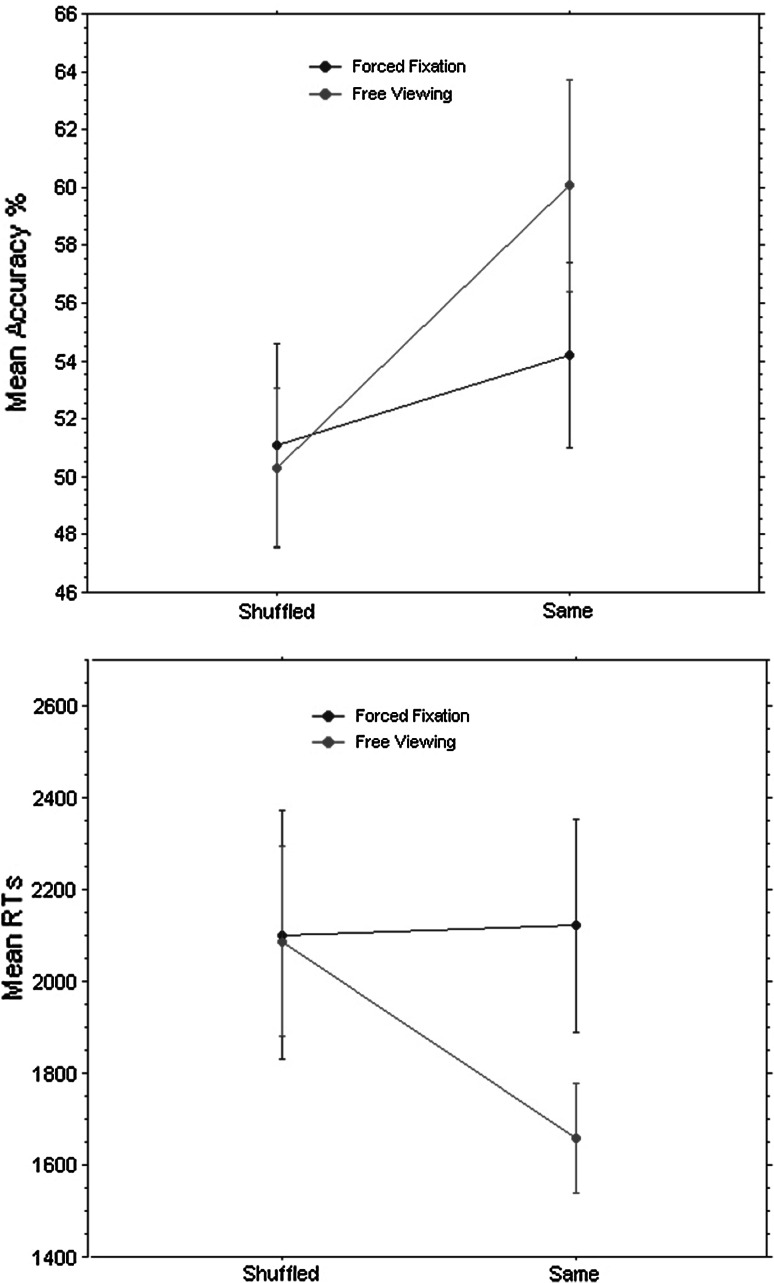
Mean percentage accuracy (*top panel*) and mean RTs (*bottom panel*) in ‘shuffled’ and ‘same’ sequences shown separately for the forced fixation and free viewing conditions. The *bars* represent the 95 % confidence intervals according to Loftus and Masson’s formula (1994)

The accuracy and RT data were computed by means of SMI BeGaze^®^ analysis software for each participant. A repeated-measures 2 × 2 ANOVA on the mean percentages of accuracy was conducted with Condition (forced fixation and free viewing) and Sequence (same and shuffled) as within-subjects factors. This analysis revealed a main effect of Sequence in the task, *F*(1, 27) = 5.207, *p* = .03 (see Fig. 3). Additionally, accuracy was significantly above chance when patterns were viewed according to the ‘same’ sequence in the free viewing condition, *t*(1, 27) = 2.751, *p* = .01, and not when the sequence was ‘shuffled’ (in the same condition), *t*(1, 27) = .102, *p* = .92.

Similarly as for the accuracy data, a repeated-measures 2 x 2 ANOVA for the mean RTs was performed with Condition (forced fixation and free viewing) and the Sequence (same and shuffled) as within-subjects factors. The analysis revealed an interaction effect of Condition * Sequence, *F*(1, 27) = 4.224, *p* = .04 (see Fig. 3). Paired samples *t* test on mean response times revealed significant difference between ‘same’ and ‘shuffled’ sequence in the free viewing condition (with longer response times in the latter comparing to the former sequence), *t*(1, 27) = −2.356, *p* = .026, but not in the forced fixation condition, *t*(1, 27) = .131, *p* = .896.

### Pupillary results

We also obtained pupillary measurements as an estimate of mental effort (Kahneman 1973; Kahneman and Beatty 1966; see also Alnæs et al. 2014) in the two conditions, since some authors have argued that memory could be better in free viewing than forced fixation, because the latter condition may be more taxing for attentional capacity (cf. Martarelli and Mast 2013).

Because each square covered around 6° of visual angle and the amplitude of pupillary light reflexes within the region of 15° of visual angle is greatest at the center region which is of about 6° (Mizukawa 2009), we excluded those trials in which (159 out of 754 trials) a black square appeared at central fixation and computed mean pupillary changes in the forced fixation (mean pupillary change = −.213; SD = .43) and free viewing (mean pupillary change = −.287; SD = .43) and conducted a repeated-measures 2 × 3 ANOVA with Condition (forced fixation and free viewing) and Sequence (same, shuffled or novel) as within-subject factors on pupillary changes in the rest of the trials (*N* = 595). This analysis did not reveal any significant effects of Condition, *F*(1, 23) = 0.898, *p* = .353 or Sequence, *F*(2, 22) = 2.868, *p* = .079 on pupillary changes. There was no significant interaction of Condition * Sequence, *F*(2, 22) = 2.276, *p* = .126. Therefore, we found no evidence that forcing fixations taxed either working memory or attention more than moving the eyes freely.

## Discussion and conclusions

In the current study, visuospatial long-term memory for checkerboard-like patterns was facilitated through the enactment of the similar sequences of eye movements as indicated by recognition accuracy and faster response times in the ‘same’ sequence only in the free viewing but not in the forced fixation condition. This supports the hypothesis about a functional role of eye movements in visual memory (Laeng and Teodorescu 2002) and is consistent with the studies that pointed to the important role of re-fixations into the original locations from encoding (e.g., Foulsham and Kingstone 2012; Holm and Mäntylä 2007; Johansson et al. 2012; Johansson and Johansson 2014; Laeng et al. 2014; Mäntylä and Holm 2006; Valuch et al. 2013). These findings also support Hebb’s (1968) account that was at the foundations of the scanpath theory (Noton and Stark 1971a, b). Importantly, the current study investigated the space-time aspect of the eye movements’ involvement in visual memory, which has been disregarded in previous studies (e.g., Brandt and Stark 1997; Laeng and Teodorescu 2002). In conclusion, not just re-fixations to the original locations but also the temporal sequences of the eye movements do play a crucial role in long-term visuospatial memory.
